# Supplementary figures and images for: Flavor Profile of Tomatoes Across Different Cultivation Times Based on GC × GC-Q/TOFMS
Source: Foods. 2025 Aug 26;14(17):2975. doi: 10.3390/foods14172975 (PMC12428567; doi:10.3390/foods14172975)

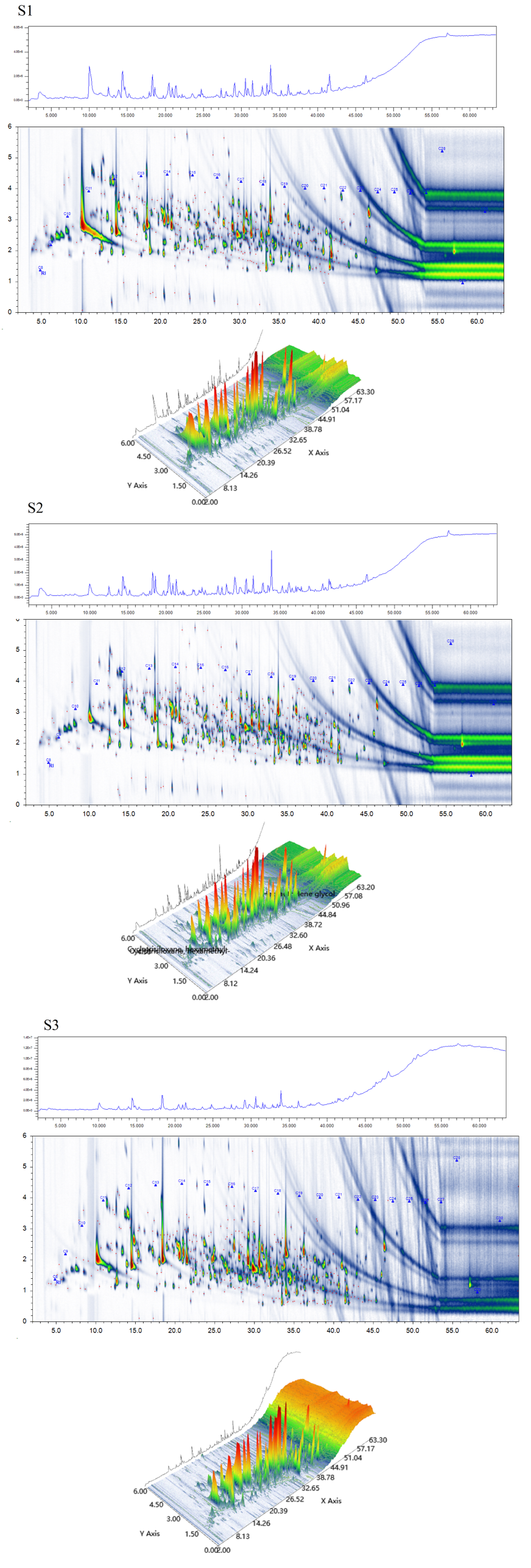

Supplement: Supplementary file 1 [file foods-14-02975-s001.zip › foods-3740931-Figure S1.JPG]
